# Splenic contraction associated with sudden, transient hypotension and sympathoexcitation in humans

**DOI:** 10.1113/EP093761

**Published:** 2026-07-17

**Authors:** Angelica Lodin‐Sundström, Gabriel Axelsson, Lisa Ljungberg, Mats H. Linér, Bodil Sjögreen, Johan P. A. Andersson

**Affiliations:** ^1^ Department of Experimental Medical Science Lund University Lund Sweden; ^2^ Department of Biology Lund University Lund Sweden; ^3^ Department of Health Sciences Mid Sweden University Sundsvall Sweden

**Keywords:** blood pressure, cardiovascular response, spleen, sympathetic activity

## Abstract

The spleen is traditionally recognised for its roles in immunity and blood filtration, and it is known to contract and release stored erythrocytes in hypoxic conditions. Recent findings suggest that the spleen may also contribute to cardiovascular regulation. This study investigated whether the spleen contracts during conditions that activate the sympathetic nervous system, including transient hypotension and psychophysiological stress responses. Eighteen healthy volunteers underwent four interventions known to be associated with either unloading of the arterial baroreceptors or sympathoexcitation: head‐up tilt, cold‐pressor test, thigh‐cuff release and maximal‐duration apnoea. Splenic volume was assessed repeatedly using ultrasound imaging, and cardiovascular variables were recorded continuously. All interventions elicited significant reductions in splenic volume (head‐up tilt −5.3%, *P* = 0.0386; cold‐pressor test −6.6%, *P* < 0.001; thigh‐cuff release −11.9%, *P* = 0.00118; apnoea −9.3%, *P* = 0.00159; Wilcoxon's signed rank test vs. baseline), indicating that splenic contraction occurs not only during hypoxic conditions (apnoea) but also in response to baroreceptor unloading (head‐up tilt, thigh‐cuff release) and general sympathetic activation (cold‐pressor test). These findings suggest that the spleen may act as a constitutive effector of the sympathetic nervous system, possibly contributing to short‐term blood pressure regulation. The results highlight a previously underappreciated role of the spleen in cardiovascular homeostasis and raise new questions about its contribution to systemic vascular control and stress physiology.

## Abstract (highlights)

- **What is the central question of this study?**
  Does the human spleen contract during transient hypotension and generalised sympathoexcitation, implying that the spleen is a constitutive effector of the sympathetic nervous system?
- **What is the main finding and its importance?**
  The human spleen contracts in response to all four tested interventions involving sympathoexcitatory stimuli, including transient hypotension (head‑up tilt, thigh‑cuff release) and generalised sympathetic activation (cold‐pressor test, apnoea), suggesting that the spleen is a constitutive effector of the sympathetic nervous system and raising questions whether splenic contraction could contribute to short‐term blood pressure regulation.

## 1 INTRODUCTION

The spleen is a unique lymphoid organ in relation to the circulatory system, being highly vascularised and integrated into the systemic circulation. Referred to as the ‘lymph node of the blood’ (Isbister, 1997), it has no afferent lymphatics but rich efferent lymphatics. The spleen is primarily associated with functions related to the immune system and its capacity to filter the blood and remove old erythrocytes (Mebius & Kraal, 2005). Less widely recognised, the spleen is also capable of contracting, resulting in a release of erythrocytes stored within the spleen, which leads to temporary increases in haematocrit and haemoglobin concentration in the circulating blood (Shephard, 2016; Stewart & McKenzie, 2002). This function is provided by the action of contractile proteins in the splenic capsule, splenic trabeculae and the muscular walls of intrasplenic arterial and venous vessels (Pinkus et al., 1986). Splenic contraction is mediated by stimulation of the spleen by efferent sympathetic noradrenergic fibres (Felten et al., 1985; Mignini et al., 2003), and α_1_‐adrenergic receptors within the spleen are involved in the response (Cabanac et al., 1998; Ojiri et al., 1993). Several conditions can cause a reduction in splenic volume, secondary to splenic contraction. It has been reported that the splenic volume decreases during physiological challenges such as exercise, apnoea, exposure to either normobaric or hypobaric hypoxia, and hyperoxic hypercapnic rebreathing (Elia et al., 2022; Holmström et al., 2021; Lodin‐Sundström & Schagatay, 2010; Lodin‐Sundström et al., 2021; Persson et al., 2023; Schagatay et al., 2005, 2020; Stewart et al., 2003). In these conditions, it is plausible that the temporary increase in circulating erythrocytes would be of some physiological or ‘protective’ value. Indeed, it has been shown that splenectomised individuals have shorter maximal‐apnoea durations than control subjects (Baković et al., 2005; Schagatay et al., 2001). Following removal of the stimulus causing splenic contraction, the spleen gradually returns towards its original volume within a few minutes, and released erythrocytes seem to be resequestered and stored within the spleen (Baković et al., 2003; Schagatay et al., 2001, 2005). In addition to the conditions above, in the context of cardiac magnetic resonance imaging there is a phenomenon known as ‘splenic switch‐off’. This refers to a marked reduction in splenic perfusion and signal intensity seen on imaging after administration of adenosine (Bakula et al., 2022), which causes vasodilation of coronary and splanchnic blood vessels, but not in the spleen.

Since the spleen is located in the systemic circulation and innervated by sympathetic fibres, it is possible that it could be associated with, as of yet undiscovered, additional physiological functions. In fact, in accordance with the suggestion by Baković et al. (2013), the spleen may represent a constitutive effector of the sympathetic nervous system under stressful situations. As such, it is possible that the spleen would be able to contract and reduce its volume in conditions besides those described above, which represent conditions characterised by different forms of hypoxia and hypercapnia. We were interested in elucidating whether the spleen would contract and reduce its volume in conditions characterised by either rapid reductions in blood pressure or a general increase in efferent sympathetic activity, especially by sympathetic activity known to induce vasoconstriction in the systemic circulation. We hypothesised that the spleen would be able to reduce its volume secondary to a reduction in blood pressure, possibly indicating a role for the spleen in short‐term blood pressure regulation, and that splenic contraction would follow general sympathoexcitation. To investigate this hypothesis, we used four interventions known to cause either transient hypotension, which would involve countermeasures following activity of the arterial baroreceptor reflex, or sympathoexcitation and associated systemic vasoconstriction. The first intervention was a passive transition from a head‐down to a head‐up position. The second intervention was the so called cold‐pressor test (CPT), during which one hand and forearm are immersed into water containing ice. The third intervention involved the inflation of thigh cuffs to a suprasystolic blood pressure for 3 min, followed by their sudden deflation. Finally, the fourth intervention was apnoea for maximal, individual duration. Both the head‐up transition and the thigh‐cuff deflation induce transient systemic hypotension, which is counteracted by reflexes involving the arterial baroreceptors and increased activity of efferent sympathetic fibres to the heart and arterial resistance vessels (Hildebrandt et al., 1979; Panerai et al., 2015; van Zanten et al., 2024). The CPT causes an increase in sympathetic activity (Lamotte et al., 2021), without a reduction in arterial blood pressure. Instead, the sympathetically induced increase in cardiac activity and peripheral vasoconstriction increase blood pressure above baseline (Andersson et al., 2000). Apnoea is known to be associated with a simultaneous increase in both parasympathetic vagal stimulation of the heart, causing bradycardia, and sympathetic stimulation of blood vessels, causing peripheral vasoconstriction (Fagius & Sundlöf, 1986; Finley et al., 1979; Gooden, 1994). The reduction in cardiac output does not match the increase in vascular resistance, and as a consequence the blood pressure increases with increased duration of a breath‐holding episode (Persson et al., 2023).

The aim of this study was to elucidate to what extent the interventions described above would elicit splenic contraction, assessed using repeated sonographic imaging of the spleen. Assuming that the spleen represents a constitutive effector of the sympathetic nervous system, we hypothesised that the spleen would contract in all these conditions.

## 2 METHODS

### 2.1 Ethical approval

This study was reviewed and approved by the Swedish Ethical Review Authority (2023‐05645) and conformed to the standards set by the latest version of the *Declaration of Helsinki*, except for registration in a database. All participants gave written consent after being provided with a detailed written and verbal explanation of the study procedures.

### 2.2 Participants

Nineteen healthy volunteers (5 women, 14 men) were recruited for the study. It was decided that one male volunteer should not perform one of the trials included in the protocol (the thigh‐cuff manoeuvre (TCM)) due to a history of vascular surgery that was unknown to the experimenters prior to recruitment. Thus, the final group of participants included eighteen healthy volunteers (5 women, 13 men). Their mean (SD, range) age was 29 (11, 21–52) years, height 181 (8, 165–194) cm and body mass 79 (14, 54–106) kg. Exclusion criteria were age below 18 or above 55 years, any known acute or chronic disease (including cardiovascular, respiratory, metabolic, endocrine, neurological or infectious conditions), splenectomy, any usage of drugs except for contraceptives, and pregnancy or attempting to become pregnant. Anomalies in blood pressure, lung function or electrocardiogram (ECG), measured and evaluated before beginning any trials, would lead to discontinuation of further participation.

### 2.3 Experimental protocol

Upon arrival at the laboratory (ambient conditions: air temperature 22.3 (1.0)°C, barometric pressure 755 (10) mmHg, relative humidity 32 (15)%), the participant received verbal instructions on the experimental protocol and equipment. After being encouraged to ask any questions about the protocol, the participant signed an informed consent form and completed a health questionnaire. Measurements of height, body mass, blood pressure and forced spirometry (both upright and supine) were performed, followed by a recording of a 12‐lead ECG. Once cleared for further participation, the participant assumed an upright, standing position being fastened to a tilt‐table. By removing the shirt, the left thoracoabdominal, dorsolateral area of the participant was exposed to allow repeated ultrasound imaging of the spleen. Non‐invasive probes for instruments used during the experimental session (see below) were attached to record continuous cardiovascular data and arterial oxygen saturation. The protocol involved subjecting the participant to four different trials: 1) head‐up tilt (HUT), 2) cold‐pressor test (CPT), 3) thigh‐cuff manoeuvre (TCM), and 4) apnoea test (APN). Each trial was performed in duplicates, and trials were separated by 5–10 min to allow recovery from the previous trial and transitions between positions for the different trials.

The first trial was a passive HUT trial. The participant, being fastened to the tilt‐table, was declined at a supine −15‐degree head‐down tilt position for 5 min. Following that, a +45‐degree HUT position was sustained for 5 min before repeating the procedure once more. After completing the two HUT trials, the participant moved from the tilt‐table to a chair positioned next to an arm‐immersion container, containing cold water and ice, to perform the CPT trial. The water temperature was 0.8 (0.6)°C. Following 5 min of rest on the chair and 2 min of baseline recordings, the participant's right hand and forearm was immersed in the container for 1 min. Immediately following the end of immersion, the hand and forearm was dried and kept warm under a dry towel. The first and second immersion periods were separated by 5 min of rest. After the CPT, the participant transitioned to a bed on which the supine position was assumed. During a 10 min rest period, a thigh cuff was positioned as proximal as possible on each thigh, that is, two cuffs were used for each participant. After the rest period, the TCM trial ensued. This involved rapidly pressurizing the cuffs to exceed the systolic blood pressure by 20–40 mmHg. The suprasystolic cuff pressure was maintained for 3 min, and during this period the cuff pressure was adjusted when needed according to any change in continuously recorded systolic blood pressure. After 3 min of inflation, the pressure was abruptly released so that both cuffs were rapidly and simultaneously deflated, followed by a 5 min rest period before the second attempt started. Finally, 5 min after the second TCM, the participant performed the two APN trials. The participant was directed to avoid hyperventilation and maintain a state of relaxation throughout the APN trials. A nose‐clip was applied 30 s before the APN. Ten seconds before the APN, the participant exhaled maximally, reaching residual volume. Immediately after this, the participant inhaled ambient air from a pre‐filled rubber bladder containing a volume of ambient air equivalent to 85% of the supine vital capacity. The completed inhalation of this volume of air marked the start of the APN. The participant attempted a maximal‐duration APN. After completing the first APN, the participant rested for 5 min in the supine position before repeating the procedure once more.

### 2.4 Equipment and measurements

To measure height and body mass, a wall‐mounted height measurer and an electric scale (BF214, Omron Healthcare Europe, Hoofddorp, Netherlands) were used. Pre‐trial blood pressure in the seated, resting position, was measured using an automatic sphygmomanometer (Boso‐medicus, Bosch + Sohn GMBH, Jungingen, Germany). A hand‐held spirometer (Micro Plus, Micro Medical Ltd, Rochester, UK) was used for forced spirometry. An ECG‐monitor (Cardiovit AT‐1 G2, Schiller, Doral, FL, USA) was used for recording the 12‐lead ECG.

Heart rate (HR), stroke volume (SV), cardiac output (CO), total peripheral resistance (TPR), as well as systolic, mean and diastolic arterial blood pressures (SBP, MAP, DBP) were recorded continuously using a finger photoplethysmograph (Finapres NOVA, Finapres Medical Systems BV, Enschede, The Netherlands). The Finapres recorded finger arterial pressure using a finger cuff, that was placed on the middle phalanx of the left middle finger, with a built‐in photoplethysmograph, calibrated using a blood pressure arm cuff placed over the left brachial artery. The finger pressure was reconstructed into brachial arterial pressure. The Finapres NOVA uses the Modelflow^®^ algorithm to calculate cardiovascular variables, such as SV, CO and TPR, from the recorded arterial pressure. Finger photoplethysmography is an indirect method for measuring cardiovascular variables, which makes it susceptible to errors (Bogert & van Lieshout, 2005; Wesseling et al., 1993). However, being non‐invasive, the method induces minimal discomfort or stress for the subjects. A stress‐free environment was desirable during the trials, to avoid any influence of the experimental conditions or measurements on the results. The arterial haemoglobin oxygen saturation ($S_{aO_{2}}$) was recorded continuously using a finger pulse oximeter (Biox 3700e, Ohmeda, Madison, WI, USA), with the probe placed on the left index finger. As a safety precaution (Lindholm & Lundgren, 2006; Linér & Andersson, 2009), one of the experimenters continuously monitored the pulse oximeter during the APN trials so that it could be interrupted if the $S_{aO_{2}}$ was to reach the predetermined termination level of 60%. The recordings of cardiovascular variables and $S_{aO_{2}}$ began 2 min prior to the first trial and were continuously run until 2 min after the end of the last trial using a data acquisition system (MP100 hardware and AcqKnowledge software, BIOPAC Systems, Goleta, CA, USA), and the data were stored for later analysis.

Ultrasound imaging of the spleen was performed using an ultrasonic device (Sonosite M‐Turbo, FUJIFILM Sonosite Inc., Bothell, WA, USA), with a curvilinear transducer (C60x/5‐2 MHz, Transducers, Sonosite Inc.). Ultrasound imaging of the spleen was performed by an experienced sonographer (A.L.S.) at 120 and 60 s before the initiation of HUT, CPT and APN, as well as 120 and 60 s before applying the thigh‐cuff pressure in the TCM trial. These pre‐trial measurements were used for determining the baseline splenic volume. For the HUT trial, splenic imaging was performed 15 and 60 s after the participant had been transitioned to the head‐up position. During the CPT, splenic imaging was performed 40 s after cold‐water immersion was initiated. For the TCM trial, images were collected at 15 and 60 s after the deflation of the thigh cuffs. Finally, for the APN trial, splenic imaging was performed 15 and 60 s after the end of breath‐hold. Two images were taken at each time point to determine maximal length (*L*), maximal thickness (*T*) and maximal width (*W*) of the spleen. Splenic volume was subsequently calculated from the collected measurements of *L*, *T* and *W* using the Pilström equation (Lodin‐Sundström, 2015): *L*π(*WT* − *T* ^2^)/3.

### 2.5 Data analysis

For the HUT trial, the baseline cardiovascular data was calculated as the mean from the period 60–30 s before changing from the head‐down to the head‐up position. For the CPT trial, the baseline data were calculated as the mean from the period 60–30 s before the immersion was initiated. For the TCM trial, the baseline data were calculated as the mean from the period 60–30 s before deflating the thigh cuffs. Finally, for the APN trial, the baseline data were calculated as the mean from the period 60–30 s before initiating the breath‐hold. To extract relevant intervention data, the following trial data was collected. For the HUT trial, the maximal change for each variable was identified from the period 0–30 s immediately after reaching the head‐up position. The mean of each variable was also calculated from the period 30–60 s following head‐up transition. For CPT, the maximal change for each variable was identified from the period 0–30 s immediately following initiation of immersion. The mean for each variable from the last 30 s of immersion was also calculated. For TCM, the maximal change for each variable was identified from the period 0–30 s immediately after deflating the thigh cuffs. The mean of each variable was also calculated from the period 30–60 s following cuff deflation. For APN, the mean of each variable was calculated from the last 30 s of the breath‐holding period. For each participant, a mean value of baseline and intervention data, respectively, from the two trials of the same type was calculated. For each intervention, the relative change from baseline was also calculated for each variable for the means calculated for the trial periods described above.

The mean splenic volume from the two imaging time points representing the baseline before each trial was calculated for each participant. For trials in which duplicate splenic imaging had been performed during or after the actual intervention (HUT, TCM and APN), a mean of the two splenic volumes obtained was calculated. For CPT, the splenic volume obtained from the images collected 40 s after cold‐water immersion was initiated was used as the intervention splenic volume. Both absolute splenic volumes and relative changes from baseline during the trials were analysed and compared between trials.

Data were checked for normal distribution, using the Shapiro–Wilk test, before further statistical tests were performed. All variables, besides TPR and splenic volume, were normally distributed. Baseline data for the different trials were compared using either one‐way repeated measures analysis of variance (ANOVA), with consideration of Mauchly's test of sphericity, or the non‐parametric related‐samples Friedman's two‐way ANOVA by ranks (Friedman's test; TPR and splenic volume). For all variables except TPR and splenic volume, values for the trial periods were compared to baseline using the two‐tailed, Student's paired *t*‐test. For TPR and splenic volume, the related‐samples Wilcoxon's signed rank test was used to assess differences between baseline and trial data. Furthermore, the absolute splenic volumes for the different trials were compared using Friedman's test with Bonferroni‐corrected Wilcoxon's signed rank test. The relative change in splenic volume from baseline was normally distributed for all trials. Therefore, the relative changes in splenic volume among the different trials were compared using a one‐way repeated measures ANOVA with consideration of Mauchly's test of sphericity. Pearson's correlation analysis was conducted to examine the linear association between relative changes in TPR and relative changes in splenic volume, and correlation coefficients (*r*) were calculated. For all statistical analysis, IBM SPSS Statistics, Version 30.0.0.0 (IBM Corp., Armonk, NY, USA) was used. The level used for accepting significance was *P* < 0.05, and exact *P*‐values are stated in the text and figures, unless *P* < 0.001. Values reported in the text are means (SD), unless otherwise stated.

## 3 RESULTS

Baseline values for the recorded cardiovascular variables and the splenic volume for the different trials are presented in Table 1. Although the baseline values for the cardiovascular variables did differ among the different tests, presumably due to, for example, differences in body positions and anticipatory responses, the splenic volume before the different trials did not differ. Baseline splenic volume displayed excellent reliability between conditions, assessed using the intraclass correlation coefficient (ICC = 0.930, *P* < 0.001, coefficient of variation (CV%) = 10.5 (3.6)%), in agreement with previous reporting (Holmström et al., 2022). Absolute reliability for splenic volume was quantified using the smallest real difference (SRD), representing the minimum change required to exceed measurement error. The SRD was calculated as 1.96 × √2 × standard error of measurement (SEM), where SEM = SD × √(1 − ICC). This resulted in a SRD of 13 mL. The responses elicited by each trial are compared to the specific trial's baseline values.

**TABLE 1:** Baseline values for cardiovascular variables and splenic volume.

Variable	HUT	CPT	TCM	APN	*P*
Heart rate (beats min^−1^)	60 (7)	73 (12)	61 (8)	70 (10)	<0.001
Stroke volume (mL)	98 (22)	79 (19)	95 (22)	103 (25)	<0.001
Cardiac output (L min^−1^)	5.9 (1.3)	5.7 (1.2)	5.7 (1.2)	7.1 (1.7)	<0.001
Total peripheral resistance (mmHg min L^−1^)	13 (7)	17 (12)	15 (9)	12 (10)	<0.001
Systolic blood pressure (mmHg)	119 (15)	134 (13)	122 (15)	126 (15)	<0.001
Mean arterial blood pressure (mmHg)	85 (11)	103 (10)	90 (13)	92 (12)	<0.001
Diastolic blood pressure (mmHg)	67 (10)	85 (9)	71 (11)	72 (11)	<0.001
Splenic volume (mL)	249 (116)	239 (121)	241 (116)	244 (119)	0.675

*Note*: Data are presented as means (SD). *n* = 18 for all variables. All variables, besides total peripheral resistance and splenic volume, were compared across trials using one‐way repeated measures ANOVA. For total peripheral resistance and splenic volume, the non‐parametric Friedman's test was used. Abbreviations: APN, apnoea test; CPT, cold‐pressor test; HUT, head‐up tilt; TCM, thigh‐cuff manoeuvre.

### 3.1 HUT

**FIGURE 1:**
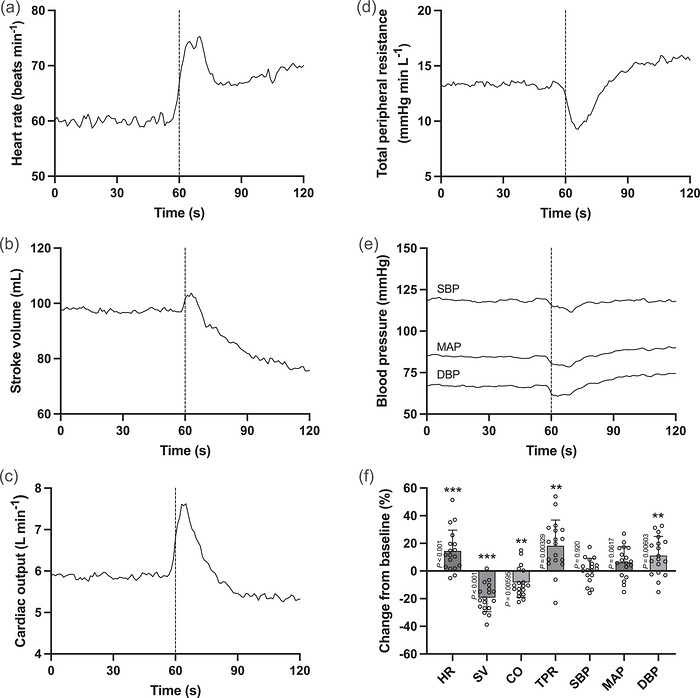
Cardiovascular responses before and after the head‐up tilt. (a–e) The mean absolute heart rate (HR), stroke volume (SV), cardiac output (CO), total peripheral resistance (TPR), and systolic, mean arterial and diastolic blood pressures (SBP, MAP, DBP) for all participants (*n* = 18) before (0–60 s) and after (60–120 s) the transition from the head‐down to the head‐up position. The vertical dashed line indicates the time of transition. (f) The relative change from baseline during the period 30–60 s after the head‐up tilt (90–120 s) for all participants. Data are presented as means with SD and individual values (*n* = 18). Absolute baseline and trial data were compared using either two‐tailed, paired *t*‐test (HR, SV, CO, SBP, MAP, DBP) or related‐samples Wilcoxon's signed rank test (TPR). *P*‐values for each dataset are shown to the left of each bar, with asterisks above bars indicating significance. ^**^ *P* < 0.01, ^***^ *P* < 0.001.

To elicit an arterial baroreceptor reflex, we exposed the participants to HUT (Figure 1). Immediately after the head‐up position was established, there was a transient reduction in blood pressure for about 15 s (Figure 1e). During this hypotensive period, the lowest SBP, MAP and DBP were 106 (14), 74 (10) and 58 (10) mmHg, respectively (all *P* < 0.001 vs. baseline, Student's paired *t*‐test). This was associated with transient increases in HR, SV and CO (Figure 1a–c). The peak values for these variables during this period were HR 80 (11) beats min^−1^, SV 108 (23) mL and CO 8.0 (1.7) L min^−1^ (all *P* < 0.001 vs. baseline, Student's paired *t*‐test). Whereas the higher HR was maintained for the duration of the analysed period, the increases in SV and CO were quickly reversed. We also observed a gradually developing vasoconstriction, as indicated by an increase in TPR over time (Figure 1d). During the period 30–60 s following the head‐up transition (Figure 1f), the SV and CO were reduced compared to baseline (SV *P* < 0.001, CO *P* = 0.00595, Student's paired *t*‐test), whereas the HR, TPR and DBP were all increased compared to baseline (HR *P* < 0.001, Student's paired *t*‐test; TPR *P* = 0.00329, Wilcoxon's signed rank test; DBP *P* = 0.00603, Student's paired *t*‐test). The SBP and MAP did not differ from baseline during this period (SBP *P* = 0.920, MAP *P* = 0.0617, Student's paired *t*‐test).

### 3.2 CPT

**FIGURE 2:**
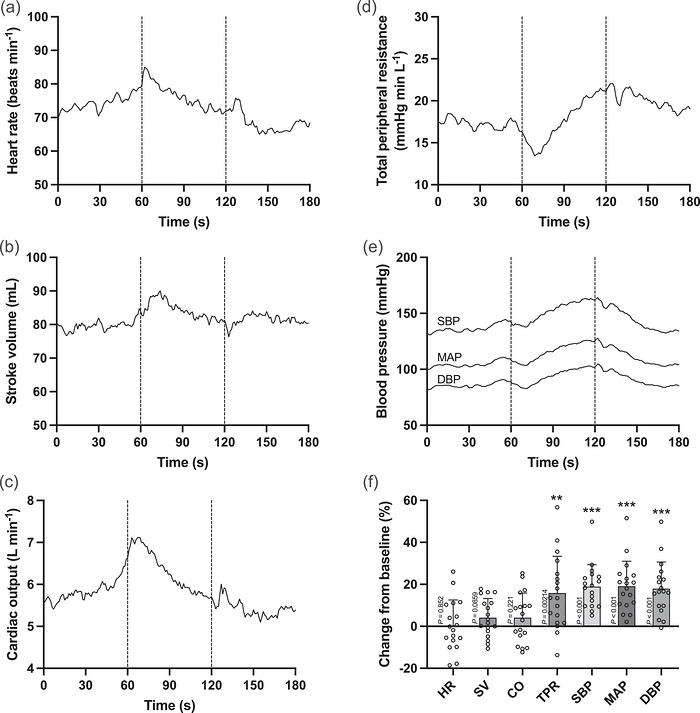
Cardiovascular responses before, during and after the cold‐pressor test. (a–e) The mean absolute heart rate (HR), stroke volume (SV), cardiac output (CO), total peripheral resistance (TPR), and systolic, mean arterial and diastolic blood pressures (SBP, MAP, DBP) for all participants (*n* = 18) before (0–60 s), during (60–120 s) and after (120–180 s) the cold pressure test. The vertical dashed lines indicate the start and end of the immersion period. (f) The relative change from baseline during the period 30–60 s into the immersion (90–120 s) for all participants. Data are presented as means with SD and individual values (*n* = 18). Absolute baseline and trial data were compared using either two‐tailed, paired *t*‐test (HR, SV, CO, SBP, MAP, DBP) or related‐samples Wilcoxon's signed rank test (TPR). *P*‐values for each dataset are shown to the left of each bar, with asterisks above bars indicating significance. ^**^ *P* < 0.01, ^***^ *P* < 0.001.

To elicit sympathetic activity without any initial reduction in arterial blood pressure, we exposed the participants to CPT (Figure 2). During the initial 30 s of immersion, there were increases from baseline in HR, SV, CO, SBP, MAP and DBP (Figure 2a–c, e). The peak values for these variables during this period were HR 90 (18) beats min^−1^, SV 95 (21) mL, CO 7.7 (2.0) L min^−1^, SBP 158 (14) mmHg, MAP 120 (14) mmHg and DBP 98 (13) mmHg (all *P* < 0.001 vs. baseline, Student's paired *t*‐test). The increases in the cardiac variables were gradually reversed as the immersion period progressed. However, during the CPT, similar to in the HUT, we observed a gradually developing vasoconstriction indicated by a progressively increased TPR (Figure 2d). During the period 30–60 s following initiation of immersion (Figure 2f), the HR, SV and CO did not differ from baseline (HR *P* = 0.852, SV *P* = 0.0859, CO *P* = 0.221, Student's paired *t*‐test), whereas the TPR, SBP, MAP and DBP were all increased compared to baseline (TPR *P* = 0.00214, Wilcoxon's signed rank test; SBP/MAP/DBP *P* < 0.001, Student's paired *t*‐test).

### 3.3 TCM

**FIGURE 3:**
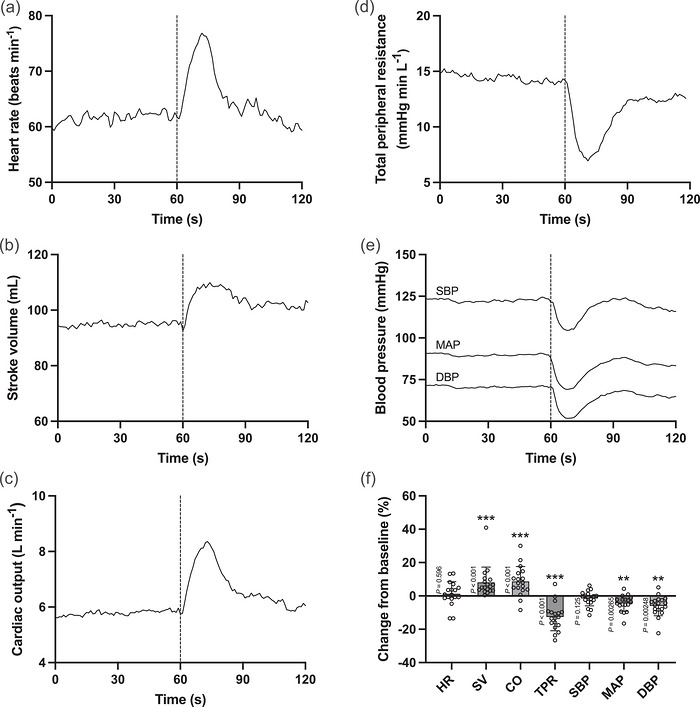
Cardiovascular responses during the thigh‐cuff manoeuvre. (a–e) The mean absolute heart rate (HR), stroke volume (SV), cardiac output (CO), total peripheral resistance (TPR), and systolic, mean arterial and diastolic blood pressures (SBP, MAP, DBP) for all participants (*n* = 18) before (0–60 s) and after (60–120 s) the release of the thigh‐cuff pressure. The vertical dashed line indicates the time of deflation. (f) The relative change from baseline during the period 30–60 s after the thigh‐cuff deflation (90–120 s) for all participants. Data are presented as means with SD and individual values (*n* = 18). Absolute baseline and trial data were compared using either two‐tailed, paired *t*‐test (HR, SV, CO, SBP, MAP, DBP) or related‐samples Wilcoxon's signed rank test (TPR). *P*‐values for each dataset are shown to the left of each bar, with asterisks above bars indicating significance. ^**^ *P* < 0.01, ^***^ *P* < 0.001.

Similar to the HUT, the TCM was used to elicit an arterial baroreceptor reflex in the participants (Figure 3). Immediately upon release of the cuff pressure, there were marked and expected cardiovascular changes. The TPR and blood pressure rapidly decreased during the initial 30 s (Figure 3d–e). During this period, the lowest values for each of these variables were TPR 7 (4) mmHg min L^−1^, SBP 103 (10) mmHg, MAP 68 (8) mmHg and DBP 51 (8) mmHg (all *P* < 0.001 vs. baseline; TPR, Wilcoxon's signed rank test; SBP/MAP/DBP, Student's paired *t*‐test). During the same period the HR, SV and CO all increased from baseline (Figure 3a–c). Peak values for these variables were HR 79 (10) beats min^−1^, SV 115 (24) mL and CO 8.6 (1.9) L min^−1^ (all *P* < 0.001 vs. baseline, Student's paired *t*‐test). During the period 30–60 s following the cuff‐pressure release (Figure 3f), the HR and SBP did not differ from baseline (HR *P* = 0.596, SBP *P* = 0.125, Student's paired *t*‐test), but the TPR, MAP and DBP were still reduced (TPR *P* < 0.001, Wilcoxon's signed rank test; MAP *P* = 0.00265, Student's paired *t*‐test; DBP *P* = 0.00248, Student's paired *t*‐test), while the SV and CO were increased (both *P* < 0.001, Student's paired *t*‐test).

### 3.4 APN

**FIGURE 4:**
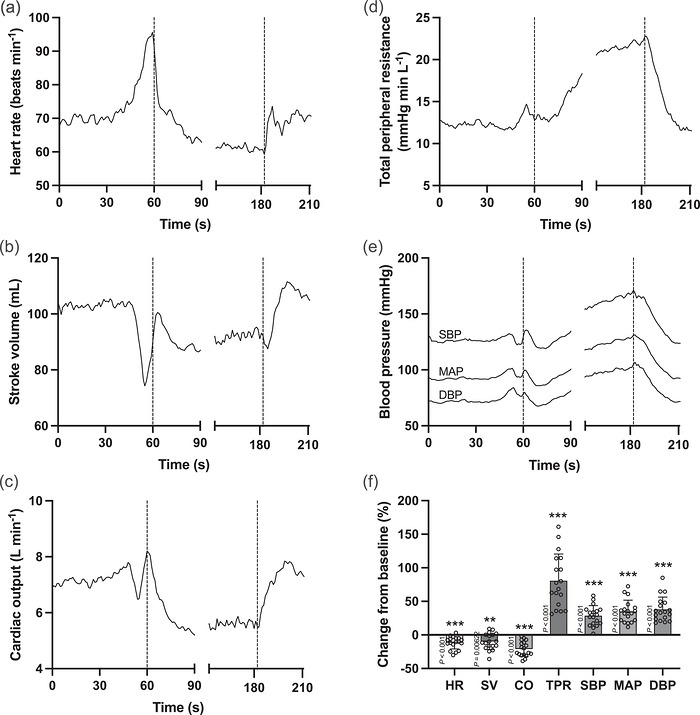
Cardiovascular responses before, during and after the apnoea test. (a–e) The mean absolute heart rate (HR), stroke volume (SV), cardiac output (CO), total peripheral resistance (TPR), and systolic, mean arterial and diastolic blood pressures (SBP, MAP, DBP) for all participants (*n* = 18) before (0–60 s), during (60–181 s) and after (181–211 s) the apnoea. The vertical dashed lines indicate the start and end of the apnoea. Breaks in the lines reflect the fact that apnoea durations varied among the participants, and the position of the end of apnoeas in the graphs has been adjusted for each participant to match the mean duration of the apnoeas (121 s). (f) The relative change from baseline during the last 30 s of apnoea for all participants. Data are presented as means with SD and individual values (*n* = 18). Absolute baseline and trial data were compared using either two‐tailed, paired *t*‐test (HR, SV, CO, SBP, MAP, DBP) or related‐samples Wilcoxon's signed rank test (TPR). *P*‐values for each dataset are shown to the left of each bar, with asterisks above bars indicating significance. ^**^ *P* < 0.01, ^***^ *P* < 0.001.

The APN trial was used because of its well‐documented ability to elicit sympathetic stimulation of blood vessels and the spleen. The mean APN duration was 121 (42) s with a range of 73–221 s. Typical cardiovascular responses were initiated during APN (Figure 4a–e). The cardiac values were all reduced from baseline as the breath‐holding period progressed. During the last 30 s of the APN (Figure 4f), the HR, SV and CO were all reduced from baseline (HR and CO *P* < 0.001, SV *P* = 0.00622, Student's paired *t*‐test). Attributable to increased sympathetic activity directed at systemic resistance vessels, during the same period the TPR, SBP, MAP and DBP were all increased from baseline (all *P* < 0.001; TPR, Wilcoxon's signed rank test; SBP/MAP/DBP, Student's paired *t*‐test).

### 3.5 Splenic volume

**FIGURE 5:**
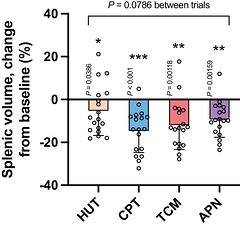
Changes in splenic volume. All trials—head‐up tilt (HUT), cold‐pressor test (CPT), thigh‐cuff manoeuvre (TCM), and apnoea test (APN)—resulted in a reduction from baseline in splenic volume. Data are presented as means with SD and individual values (*n* = 18). Absolute baseline and trial data were compared using related‐samples Wilcoxon's signed rank test. *P*‐values for each dataset are shown above each bar, with asterisks above bars indicating significance. The relative changes in splenic volumes did not differ between trials (one‐way repeated measures ANOVA). ^*^ *P* < 0.05, ^**^ *P* < 0.01, ^***^ *P* < 0.001.

As noted above, the baseline splenic volume did not differ among trials (Table 1). We observed that the splenic volume was reduced from baseline as result of all trials (Figure 5; HUT *P* = 0.0386, CPT *P* < 0.001, TCM *P* = 0.00118, APN *P* = 0.00159, Wilcoxon's signed rank test). After the HUT, the splenic volume had been reduced to 235 (110) mL. During the CPT, splenic volume was 207 (121) mL. After the TCM, splenic volume was 210 (100) mL. Finally, after the APN, the splenic volume had been reduced to 217 (89) mL. Friedman's test revealed a difference in splenic volume during or after the trials (*P* < 0.0169). The splenic volume following the HUT differed from the volumes observed in association with CPT and TCM (both *P* = 0.0402, pairwise Bonferroni‐corrected Wilcoxon's signed rank test). No other pairwise comparisons revealed any differences in splenic volume between trials. When comparing the relative changes in splenic volumes for the four trials, no differences between the trials were observed (*P* = 0.0786, one‐way repeated measures ANOVA).

For the HUT and the TCM, there were no significant correlations between the relative changes in TPR and the relative changes in splenic volume (HUT: Pearson's *r* = 0.08095, *P* = 0.7495; TCM: Pearson's *r* = 0.02841, *P* = 0.9109). However, for the CPT and the APN, there were significant, inverse correlations between the relative changes in TPR and the relative changes in splenic volume (CPT: Pearson's *r* = −0.5351, *P* = 0.0221; APN: Pearson's *r* = −0.4702, *P* = 0.0490).

## 4 DISCUSSION

This study describes novel stimuli to splenic contraction, contributing to the understanding of splenic function and its relation to increased sympathetic activity and cardiovascular dynamics. We used different interventions for initiating sympathetic activity known to induce systemic vasoconstriction. In the case of APN, it has been established that the sympathetic activity that elicits systemic vasoconstriction simultaneously causes splenic contraction (Baković et al., 2003; Lodin‐Sundström & Schagatay, 2010). However, no previous study has addressed whether the HUT, CPT or TCM could be linked to splenic contraction. In the present study, we show that the splenic volume is reduced with interventions causing sudden, transient hypotension, that is, HUT and TCM, and sympathoexcitation, that is, CPT and APN. These observations lend support to the notion that the spleen represents a constitutive effector of the sympathetic nervous system and that it has the ability to contract in a variety of conditions associated with sympathoexcitation. This raises intriguing questions about whether splenic contraction could contribute to short‐term blood pressure regulation.

### 4.1 Cardiovascular responses and sympathoexcitation

In the HUT trial, shortly after attaining the head‐up position, the SV and CO were gradually reduced from pre‐transition levels, most likely owing to a reduction in cardiac preload as the trial progressed (Dorogovtsev et al., 2021). This was accompanied by a small and transient hypotension period. The reduction in blood pressure most likely activated the arterial baroreceptor reflex, increasing efferent sympathetic signalling to the heart and systemic blood vessels (Ghosh & Pandit, 2022; O'Leary et al., 2003). The increase in efferent sympathetic signalling was reflected by the sustained tachycardic response alongside a gradual increase in systemic vascular resistance that developed in the period following the transition to the head‐up position. Thus, we can assume that the HUT trial caused sympathoexcitation via the arterial baroreceptor reflex.

While the HUT trial caused a minor reduction in blood pressure, the TCM trial was characterised by an immediate, augmented reduction in systemic blood pressure when the thigh‐cuff pressure was released, in accordance with previous observations (Panerai et al., 2015). During the inflation period, the distal parts of the lower extremities were deprived of arterial blood flow, producing ischaemia and an accumulation of metabolites that have vasodilatory effects (Rosenberry & Nelson, 2020). However, the cuffs, by completely compressing femoral arteries because of their suprasystolic pressure, cause an ‘artificial’ and immeasurable vascular resistance. The sudden reduction in peripheral resistance resulting from deflating the cuffs leads to a hyperaemic response in the legs (i.e., reactive hyperaemia) that causes a sudden drop in blood pressure (Panerai et al., 2015). Our recordings of cardiovascular changes confirm these responses in the post‐deflation period. Indicative of a general sympathoexcitation following activation of the arterial baroreceptor reflex (Ghosh & Pandit, 2022), we observed an immediate increase in HR, a sustained increase in SV and gradual restorations from the initial reductions towards baseline for TPR and blood pressure.

Whereas the HUT and TCM trials were used because of their ability to initiate sympathoexcitation via the arterial baroreceptor reflex following hypotension, the mechanisms are different during the CPT. During the immersion period, increased afferent signalling from cutaneous cold thermoreceptors and nociceptors will cause a general increase in sympathetic activity to the heart and blood vessels (Allen et al., 1992; Frey et al., 1980a; Lamotte et al., 2021). As expected, we observed initial increases in HR, SV and CO during immersion, accompanied by a gradual increase in arterial blood pressure. However, while the TPR continued to increase with time as the immersion period progressed, the cardiac variables all showed a return towards baseline levels after the initial reflex response. This may be explained partly as the result of a baroreceptor reflex stimulated by the increased arterial blood pressure (Frey et al., 1980b). Nevertheless, the TPR as well as the blood pressure increased markedly from baseline during the CPT because of the sympathoexcitation (Andersson et al., 2000; Frey et al., 1980a).

Consistent with previous studies (Andersson et al., 2025; Persson et al., 2023), the APN trial induced a marked increase in TPR, while HR, SV and CO all decreased. These cardiovascular changes to breath‐holding are the integrated outcome of several reflex mechanisms occurring simultaneously, often referred to as the diving response, with interactions between these reflexes (Gooden, 1994). The bradycardia results from increased parasympathetic activity, while the peripheral vasoconstriction is produced by increased activity of the sympathetic nerves. The apnoea‐induced vasoconstriction we observed was greater in magnitude than the simultaneous decrease in CO, resulting in a gradual increase in systemic blood pressure with duration of the APN trial. Overall, the cardiovascular changes indicate that the APN trial was associated with sympathoexcitation, in accordance with earlier observations (Fagius & Sundlöf, 1986; Leuenberger et al., 2001).

### 4.2 Splenic contraction during trials

In the HUT trial, a very modest, transient period of hypotension was elicited. This slight hypotension was associated with an absolute reduction in splenic volume that was not as pronounced as in the other trials included in the protocol, even though the relative change from baseline was similar in all trials. With the TCM, a more pronounced transient hypotensive response was recorded, associated with a noticeable reduction in splenic volume. Combined, the results from these two trials indicate that situations causing a sudden decrease in systemic blood pressure elicit splenic contraction. It seems likely that the splenic contraction could be related to an unloading of the arterial baroreceptors, increasing efferent sympathetic signalling when hypotension is induced (Ghosh & Pandit, 2022). Splenic contraction could likely be one response of increased sympathetic output from the vasomotor centre due to withdrawal of inhibitory baroreceptor activity (Palada et al., 2007), not only in these conditions but also in other situations leading to hypotension.

The sympathoexcitation associated with the CPT and APN trials also caused splenic contraction. It is established that breath‐holding causes splenic contraction (Baković et al., 2003; Lodin‐Sundström & Schagatay, 2010; Schagatay et al., 2005). Therefore, the APN trial was included in the protocol of the present study to assure that the included participants would be responsive in regard to splenic contraction. It was an interesting observation that also the CPT trial elicited splenic contraction. In this case, contrary to the HUT and TCM trials, it is not an unloading of arterial baroreceptors that causes sympathoexcitation. Rather, the afferent pathways triggering efferent sympathetic activity originate from cutaneous receptors, as described above. We report here that this type of general stress response and sympathoexcitation includes a contraction of the spleen. Again, as in the HUT and TCM trials, the observation that the spleen contracts during the CPT could be supportive of the notion that splenic contraction is a reflex response to a variety of psychophysiological stressors and not only elicited by conditions characterised by hypoxia or exercise where a release of erythrocytes would be of value. This could exemplify how the spleen may represent a constitutive effector of the sympathetic nervous system (Baković et al., 2013).

### 4.3 The spleen in blood pressure regulation

Considering the combinations of cardiovascular responses and splenic contraction elicited by the trials of the present study, it is intriguing to consider whether the spleen may have a short‐term role in the regulation of systemic blood pressure. Splenic contraction with intrasplenic vasoconstriction would contribute to an increase in TPR. Hence, the spleen could possibly act as a vascular‐resistance organ. Studies using methods such as ^111^indium‐labelled platelets, PET and MRI have reported splenic artery blood flow values ranging from approximately 65 to 168 mL min^−1^ per 100 g of tissue in healthy humans under resting conditions (Galea et al., 2019; Oguro et al., 1993; Peters et al., 1980). Assuming a mean splenic mass of about 150 g, this corresponds to a total splenic blood flow of roughly 98–252 mL min^−1^. Thus, when compared to a typical resting CO of 5 L min^−1^, the spleen receives an estimated 2–5% of the systemic arterial blood flow. These values are derived by combining published splenic perfusion measurements with standard physiological CO, as no human studies report simultaneous measurements of both variables. However, that the spleen receives approximately 5% of CO in healthy humans under resting conditions seems to be a general assumption (Isbister, 1997). Additionally, the spleen accounts for approximately 0.2% of total body mass in a mean adult (assuming 150 g relative to 70 kg). These estimates suggest that, under normal conditions, the spleen represents a small fraction of body mass yet accounts for a significant proportion of systemic circulation. It may be that having the spleen contract in situations during which an increase in systemic vascular resistance occurs would be beneficial by quickly and effectively reducing splenic perfusion while simultaneously allowing maintained perfusion of more vital tissues. It would be most interesting to have comparative blood flow measurements to different organs and the spleen in conditions such as those included in the present study to elucidate whether splenic blood flow is disproportionally reduced with sympathoexcitation. Moreover, the line of reasoning above resonates with the splenic switch‐off phenomenon, that is, a markedly reduced splenic perfusion observed with adenosine infusion (Bakula et al., 2022). Although adenosine induces vasodilation in most vascular beds via A_2_‐receptor activation (Morato et al., 2008), the spleen may exhibit reduced perfusion due to a reflex sympathetic response and systemic blood flow redistribution.

In the context of the spleen's possible role in short‐term blood pressure regulation, it is worth mentioning the splenorenal reflex (Deng & Kaufman, 2001; Hamza & Kaufman, 2004, 2009). The splenorenal reflex is a neurogenic reflex loop in which sensory signals from the spleen, following changes in splenic blood flow, influence renal sympathetic nerve activity, thereby affecting renin release, renal blood flow and systemic blood pressure. It represents a communication pathway where the spleen can modulate kidney function through coordinated afferent sensory and efferent sympathetic nerve signals, through which the spleen can exert a tonic control of systemic blood pressure and play a central role in cardiovascular homeostasis (Hamza & Kaufman, 2004, 2009). Thus, the splenorenal reflex could be operating on an intermediate to longer term in relation to blood pressure and blood volume regulation.

Accepting that the spleen has diverse roles in blood pressure regulation, it is noteworthy that a recent study addressed a potential role of the spleen in the development of arterial hypertension in humans (Nardin et al., 2025). Individuals who had undergone splenectomy were compared with those who had had a cholecystectomy (controls). No difference between the groups in the prevalence of hypertension or in the incidence of new hypertension diagnoses were found. However, individuals without a spleen showed lower 24‑h and daytime diastolic blood pressure compared with the control group. Furthermore, the study identified a significant interaction between splenectomy and the relationship between diastolic blood pressure and vascular wall cross‑sectional area, as well as capillary recruitment, suggesting that the spleen influences microvascular regulation. It was concluded that the results support a role for the spleen in human blood pressure regulation, and the authors call for larger studies to further clarify the clinical significance of these findings (Nardin et al., 2025).

### 4.4 Limitations

Several limitations should be acknowledged. To what extent the splenic contraction results in a reduction in splenic blood flow is still unknown. It is noteworthy that splenic blood flow increases above baseline in the recovery period following a CPT (Galea et al., 2019). This observation may reflect a hyperaemic compensatory response following a period of reduced splenic blood flow accompanying the splenic contraction during the CPT. In addition, some studies have demonstrated an inverse correlation between splenic perfusion and spleen size in patients with haematological disorders (Wadenvik et al., 1987) and hepatic cirrhosis (Sauter et al., 2014). Nevertheless, simultaneous investigations of splenic volume and blood flow under similar conditions as in the present study are required to provide the answer to this uncertainty. We did not assess directly the baroreceptor activity in the HUT and TCM trials, nor measure efferent sympathetic activity in any of the trials. However, it is well established that the CPT and APN elicit sympathoexcitation (Gooden, 1994; Lamotte et al., 2021). Also, in the HUT and TCM trials it is more than likely that we elicited the expected afferent and efferent autonomic reflexes (Ghosh & Pandit, 2022). This is further supported by the cardiovascular data we report for all of the trials.

The contribution of splenic contraction to cardiovascular regulation may differ across mammalian species and may be more pronounced in animals with larger spleens and larger splenic blood reservoirs. A more detailed exploration of this topic is beyond the scope of the present study.

Splenic imaging was not performed in a strict double‐blind design. However, the ultrasound operator outlining splenic dimensions did not collect or note the relevant data, that is, the length, thickness and width of the spleen, at the time of imaging. This was done by a separate experimenter that was not performing the imaging, avoiding bias.

Finger photoplethysmography is an indirect method for measuring cardiovascular variables, which makes it susceptible to errors (Bogert & van Lieshout, 2005; Wesseling et al., 1993). In comparison with intra‐arterial blood pressure measurements this technique is subject to considerable differences in absolute pressures. Despite this reservation, relative changes in pressure are closely reproduced. Furthermore, as the data extracted from the finger photoplethysmograph were primarily used for relative comparisons within subjects, the accuracy of the measurements was not as important, as potential errors are likely to be similar within the same subject.

### 4.5 Conclusions

This study demonstrates that the human spleen contracts in response to a range of sympathoexcitatory stimuli, including transient hypotension and generalised sympathetic activation. These findings expand the understanding of splenic function beyond its established roles in erythrocyte homeostasis and as a lymphoid organ. The observed reductions in splenic volume during HUT, thigh‐cuff release, hand and forearm cold stimulation, and apnoea indicate that splenic contraction is a reflexive component of systemic stress responses. Future studies combining simultaneous measurements of splenic blood flow, systemic haemodynamics and sympathetic nerve activity are warranted to clarify the magnitude and clinical significance of this mechanism, as well as any relation it may have to short‐term blood pressure regulation.

## AUTHOR CONTRIBUTIONS

Angelica Lodin‐Sundström, Mats H. Linér, Bodil Sjögreen, and Johan P. A. Andersson conceived and designed the study. All authors were involved in performing experiments. Angelica Lodin‐Sundström, Gabriel Axelsson, Lisa Ljungberg, and Johan P. A. Andersson analysed the data. All authors interpreted the results of the experiments. Angelica Lodin‐Sundström, Gabriel Axelsson, Lisa Ljungberg, and Johan P. A. Andersson drafted the manuscript. All authors were involved in the revision of the draft, approved the final version of the manuscript and agree to be accountable for all aspects of the work in ensuring that questions related to the accuracy or integrity of any part of the work are appropriately investigated and resolved. All persons designated as authors qualify for authorship, and all those who qualify for authorship are listed.

## CONFLICT OF INTEREST

The authors declare that the research was conducted in the absence of any commercial or financial relationships that could be construed as a potential conflict of interest.

## FUNDING INFORMATION

This research did not receive any specific grant from funding agencies in the public, commercial, or not‐for‐profit sectors. Funds for open access publication fees were obtained from the Lund University Library and the Faculty of Medicine, Lund University.

## GENERATIVE AI STATEMENT

Generative artificial intelligence tools have not been used in the in the preparation of this manuscript including, but not limited to, data collection and analysis, the production of tables and figures, and writing of the manuscript.

## Data Availability

Original data arising from this research are available directly from J.P.A.A. upon reasonable request.
